# Mandibular destructive radiolucent lesion: The first sign of multiple myeloma

**DOI:** 10.4317/jced.52832

**Published:** 2016-10-01

**Authors:** Eduardo-Rodrigues Fregnani, Amanda-Almeida Leite, Claudia-Joffily Parahyba, Ana-Cristina-Alo Nesrallah, Flávia-Maria-de Moraes Ramos-Perez, Danyel-Elias-da Cruz Perez

**Affiliations:** 1 Department of Oral Medicine, Hospital Sírio-Libanês, São Paulo, Brazil; 2 Department of Clinical and Preventive Dentistry, Universidade Federal de Pernambuco, Recife, Pernambuco, Brazil

## Abstract

The occurrence of a mandibular lesion as the first sign of multiple myeloma (MM) is uncommon. This report describes a case of MM diagnosed because of a mandibular lesion. A 62-year-old woman presented a destructive radiolucent lesion in the right mandibular ramus. The lesion caused rupture of the anterior cortical bone and extended from the retromolar area to the coronoid process. An incisional biopsy was performed. Histopathological examination revealed numerous pleomorphic plasma cells, some with binucleated nuclei. The tumor cells showed kappa light-chain restriction. Bone marrow biopsy showed findings of massive infiltration of neoplastic plasma cells, besides lesions in the vertebrae. The diagnosis of MM was established. The patient underwent autologous hematopoietic stem-cell transplantation. Currently, the patient is under regular follow up after 40 months of initial treatment. In conclusion, MM should be considered in the differential diagnosis of destructive mandibular lesions.

** Key words:**Mandible, multiple myeloma, radiolucent lesion.

## Introduction

Multiple myeloma (MM) is a relatively rare malignant neoplasm of plasma cells (1-6). The malignant cells are mainly characterized by the overproduction of a monoclonal kappa or lambda light chain antibody, with or without an associated heavy chain. These immunoglobulins may be found in serum or urine, and are known as M proteins in reference to multiple myeloma (4,5,7,8).

MM usually manifests as a tumor involving the marrow of several bones, and develops more commonly in the skull, vertebrae, and pelvis (2,9). The etiology is unknown, although there is an increased risk in patients exposed to ionizing radiation. In many cases, it is associated with gene rearrangements due to chromosomal translocations in the immunoglobulin heavy chain region (3).

The clinical features are related to the effects of proliferation and expansion of neoplastic plasma cells in the bone marrow, the excessive production of immunoglobulins, which often have abnormal physicochemical properties, and the suppression of normal humoral immunity. The primary manifestation is related to bone destruction induced by tumor cells (4,10). Additionally, as consequence of abnormal accumulation of light chains of immunoglobulins, approximately 15% of the patients with MM present amyloidosis. The amyloidosis may be the first sign of MM. The oral mucosa, particularly the tongue, is commonly affected by amyloidosis, which cause frequently macroglossia (3).

Histopathological analysis of bone biopsies in patients with MM have shown that increased osteoclastic activity occurs simultaneously with the development of the disease. This led to the hypothesis that local cytokines produced or induced by myeloma cells would be responsible for increased bone resorption activity. Simultaneously, because of the resorption process, the release of growth factors would increase the development of neoplastic plasma cells (11,12). Along with symptoms related to bone destruction, the patient has an increased risk of infections and fever, fatigue, anemia, nephropathy, and temporal arteritis (2,3,10,13,14).

This disease accounts for approximately 1% of all malignancies and 10% of hematologic malignancies (2,6,9,10,13,15). It mainly affects men. It is primarily a disease of the elderly, affecting individuals aged 60 to 70 years (2,6,10,13). The involvement of the maxilla and/or mandible is common, occurring in 30% of cases. However, the presence of maxillary or mandibular lesions as the first manifestation of the disease is rare (2,4,9,16). Thus, this report describes a case of multiple myeloma initially diagnosed because of a mandibular lesion.

## Case Report

A 62-year-old woman sought treatment for a mandibular lesion present for about 2 months. The patient complained of swelling at the mandible, trismus, and paresthesia on the right side of the lip and tongue. The intraoral examination showed mild edema in the retromolar region, where palpation revealed bony crepitus.

**Figure 1:**
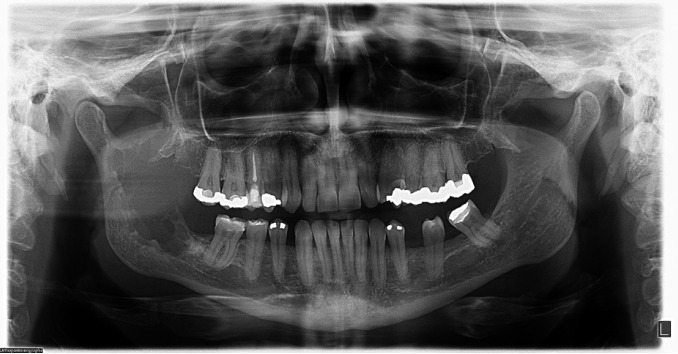
Panoramic radiograph revealing a multilocular radiolucent destructive lesion in the right mandibular ramus.

**Figure 2:**
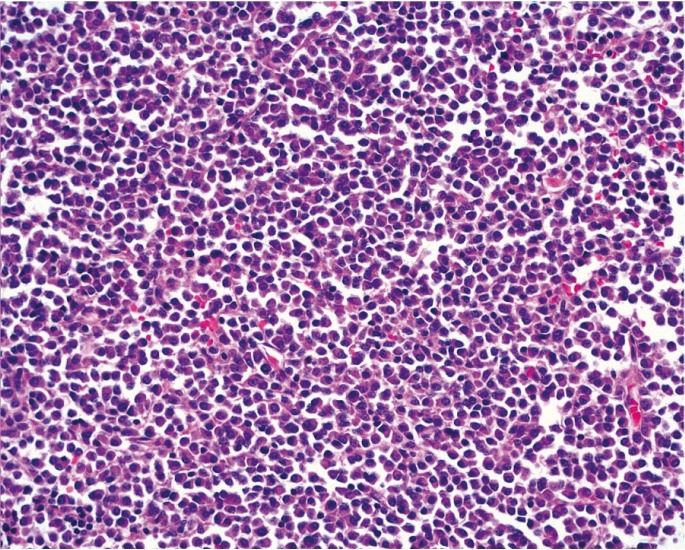
Several pleomorphic neoplastic cells with eccentric nuclei and a large eosinophilic cytoplasm (hematoxylin-eosin, x200).

**Figure 3:**
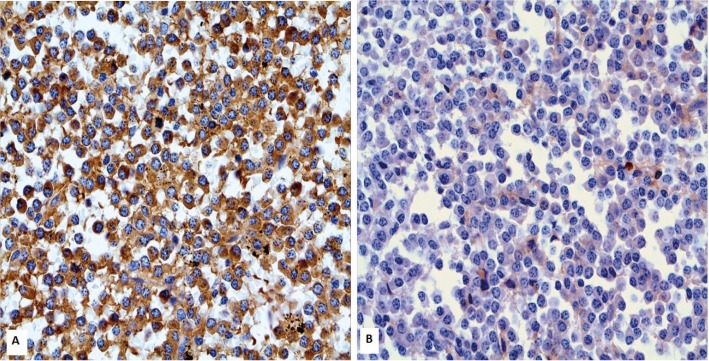
Immunohistochemistry evidenced kappa immunoglobulin light chain (A) restriction (x400).

A panoramic radiograph showed a multilocular radiolucent destructive lesion in the right mandibular ramus (Fig. 1). The lesion caused rupture of the anterior cortical bone, and extended from the retromolar area to the coronoid process. Ameloblastoma and odontogenic myxoma were the main hypotheses of diagnosis. In addition, a malignant neoplasm could not be ruled out. An incisional biopsy was performed under local anesthesia. Histopathological examination revealed numerous pleomorphic neoplastic cells with eccentric nuclei and a large eosinophilic cytoplasm (Fig. 2). Immunohistochemistry showed neoplastic cells with plasmacytic differentiation and monoclonality for kappa immunoglobulin light chains (Fig. 3), suggesting a diagnosis of plasmacytoma/MM.

The patient underwent other clinical, imaging, and laboratory tests to confirm the diagnosis. Bence Jones protein was positive in the urine. Protein electrophoresis performed with immunofixation with monospecific antisera for light and heavy immunoglobulin chains revealed the presence of a monoclonal IgG/kappa component. Bone marrow biopsy showed findings of massive infiltration of neoplastic plasma cells. In addition, the patient had diffuse lytic lesions in vertebral bodies causing cortical thinning and slight protrusion of soft tissue into the medullary cavity. Similar lesions were observed in the sacrum. Accordingly, the diagnosis of MM was confirmed. In oral cavity, macroglossia or any other signs of amyloidosis were not found.

The patient underwent chemotherapy with cyclophosphamide (50 mg), dexamethasone (40 mg), and thalidomide (100 mg). Intravenous zoledronate was administered, and 12 radiotherapy sessions (total dose: 24 Gy) were performed to control pain from the vertebral lesions. After chemotherapy, the patient underwent autologous hematopoietic stem cell transplantation (ASCT). Today, 40 months after diagnosis and initial treatment, the patient is under regular monitoring. Panoramic radiograph showed significant regression of mandibular lesion at 36 months. The patient reported persistent paresthesia only on the right side of the tongue.

## Discussion

MM is a characteristic disease of the elderly, with a slight tendency to affect men, and it is rarely diagnosed before 40 years of age. This disease is commonly disseminated, involving multiple skeletal sites, and affects mainly the skull, pelvis, vertebrae, ribs, and long bones (9,17). In this case, besides the lesion in the mandible, lesions were observed in the vertebrae. When the mandible is involved, as occurs in about 30% of cases, MM especially affects the molar region, ramus, and angle, probably because these areas exhibit strong hematopoietic activity (2,3,7,14).

Presentation in the mandible is particularly rare, and there are few reports in the literature of mandibular lesions as the initial presentation of MM. Oral manifestations may include gingival bleeding, dental pain, paresthesia, dental mobility, ulceration, and increased fullness (3,7,13,14,17,18). In this case, the clinical signs of malignancy included swelling at the site, trismus, and paresthesia.

On radiography, the appearance of MM varies. It can present as multiple well-defined, or irregular and diffuse, radiolucent lesions (2-5,17). The radiographic pattern in this case showed a destructive radiolucent lesion in the mandibular ramus associated with rupture of the anterior cortical bone. The differential diagnosis of multiple lesions with radiological findings of bony destruction includes metastatic disease, chronic osteomyelitis, and arteriovenous malformations (2,3). The differential diagnosis may also include benign odontogenic tumors, such as ameloblastoma, keratocystic odontogenic tumor, and odontogenic myxoma. Although rare in this age group, aggressive central giant cells lesions should also be included in the differential diagnosis (2).

The histopathological examination of MM shows diffuse and monotonous layers of neoplastic plasmacytoid cells, with variable degrees of differentiation that invade and replace normal host tissue. Mitotic activity can be seen with some frequency. Plasma cells of relatively normal appearance, plasmablasts with vesicular nuclear chromatin and a single visible nucleolus, or bizarre multinuclear cells may predominate (8,13). Immunohistochemistry for kappa and lambda chains is commonly performed in order to characterize the monoclonal plasma cells that are observed in this disease (12). In this case, the histopathological examination revealed numerous pleomorphic plasma cells, some of them bi-nucleate. Immunohistochemistry confirmed the diagnosis, with evidence of kappa light chain restriction.

The diagnosis of MM is based on clinical, imaging, histopathological, and laboratory findings (6). These include 10% or more monoclonal neoplastic plasma cells present on bone marrow examination, or the presence of a biopsy-proven plasmacytoma causing systemic pathology, such as anemia, hypercalcemia, multiple osteolytic lesions, and renal failure (6,10,13,15). Furthermore, the presence of more than 60% monoclonal plasma cells in the bone marrow points to the diagnosis of myeloma, without the existence of lesions in other organs (10). In this case, after the incisional biopsy and confirmation of the monoclonal neoplastic nature by immunohistochemistry, other lesions were found in the vertebrae, in addition to massive neoplastic marrow infiltration by plasma cells, and the presence of a monoclonal IgG/kappa component on protein electrophoresis.

The treatment of MM varies considerably among institutions and countries. This variation stems from the availability and cost of new drugs, and is related to different philosophies and interpretations of scientific data (6,15). In general, initial therapy is based on the consideration of ASCT. These patients should be less than 65 years of age, without other systemic limitations (6,9,15), as in this case.

The first attempt to control the disease generally consists of chemotherapy. An alkylating agent, such as melphalan, is often administered with prednisone or dexamethasone (3,9). For patients selected for ASCT, an induction regimen is devised that does not contain melphalan with prednisone as a single agent, or a combination with another agent, such as lenalidomide, is administered; ASCT follows, as an early or late approach after the first relapse (3,6,10,15). Chemotherapy with a combination of cyclophosphamide, thalidomide, and dexamethasone (19), as used in this case, has been shown to be an effective induction regimen prior to ASCT in patients with MM. Other regimens using thalidomide, bortezomib, and lenalidomide are being used for some induction therapy, maintenance therapy, and relapse phases of the disease, and may be responsible for the significant increase in the survival time of patients seen in the last decade (3,9,10,15).

For patients who have no indication for ASCT, 12-18 months of treatment with melphalan or a regimen used in ASCT patients may also be applied in this group. Inevitably, most patients will relapse, with progressive decreases in the duration of disease remission with each rescue therapy (10,15).

Bisphosphonates are used as complementary treatment, by inhibiting bone resorption and reducing pathologic fractures and hypercalcemia (3,5), as in this patient. The duration of therapy is indefinite, but some authors advocate a limitation, due to the risk of osteonecrosis of the jaw following invasive dental procedures, particularly dentoalveolar surgery. To avoid such adverse effects, the involvement of the dental surgeon is essential. Patients should be periodically monitored to maintain excellent oral health (3,14,16). In addition, radiotherapy may be used in combination with other therapies in cases with painful bone lesions. Approximately 30% of patients will need this therapy (3,11), as in this case.

The prognosis is usually unfavorable, but is variable and depends on many factors, such as the patient’s age, stage of disease, related genetic changes, and response to therapy (3,10). Patients undergoing ASCT can survive for more than 10 years, which shows that it can prolong life, but is not curative. The mean survival is 5 years, and cure has not yet been achieved (3,6). Our patient underwent ASCT, and the disease remains under control.

In conclusion, MM is an aggressive, relatively rare malignant neoplasm that usually affects the marrow at several skeletal sites. The mandible will be affected in about 30% of cases, but the initial presentation with damage to gnathic bones is particularly rare. Owing to radiographic appearance similar to other diseases, clinical and laboratory evaluation, including histopathological examination, should be done to confirm the diagnosis. In addition, the dental practitioner must be aware that MM should be considered in the differential diagnosis of mandibular destructive lesions, particularly in elderly patients.
